# ADAM19: A Novel Target for Metabolic Syndrome in Humans and Mice

**DOI:** 10.1155/2017/7281986

**Published:** 2017-02-07

**Authors:** Lakshini Weerasekera, Caroline Rudnicka, Qing-Xiang Sang, Joanne E. Curran, Matthew P. Johnson, Eric K. Moses, Harald H. H. Göring, John Blangero, Jana Hricova, Markus Schlaich, Vance B. Matthews

**Affiliations:** ^1^School of Medicine and Pharmacology, University of Western Australia, Crawley, Western Australia, Australia; ^2^Royal Perth Hospital, Perth, Australia; ^3^Department of Chemistry and Biochemistry, Florida State University, Tallahassee, FL, USA; ^4^South Texas Diabetes & Obesity Institute, University of Texas Rio Grande Valley School of Medicine, Brownsville, TX, USA; ^5^Centre for Genetic Origins of Health & Diseases, Faculty of Medicine, Dentistry & Health Sciences, University of Western Australia and Faculty of Health Sciences, Curtin University, Western Australia, Australia

## Abstract

Obesity is one of the most prevalent metabolic diseases in the Western world and correlates directly with insulin resistance, which may ultimately culminate in type 2 diabetes (T2D). We sought to ascertain whether the human metalloproteinase A Disintegrin and Metalloproteinase 19 (ADAM19) correlates with parameters of the metabolic syndrome in humans and mice. To determine the potential novel role of ADAM19 in the metabolic syndrome, we first conducted microarray studies on peripheral blood mononuclear cells from a well-characterised human cohort. Secondly, we examined the expression of ADAM19 in liver and gonadal white adipose tissue using an in vivo diet induced obesity mouse model. Finally, we investigated the effect of neutralising ADAM19 on diet induced weight gain, insulin resistance in vivo, and liver TNF-*α* levels. Significantly, we show that, in humans, ADAM19 strongly correlates with parameters of the metabolic syndrome, particularly BMI, relative fat, HOMA-IR, and triglycerides. Furthermore, we identified that ADAM19 expression was markedly increased in the liver and gonadal white adipose tissue of obese and T2D mice. Excitingly, we demonstrate in our diet induced obesity mouse model that neutralising ADAM19 therapy results in weight loss, improves insulin sensitivity, and reduces liver TNF-*α* levels. Our novel data suggest that ADAM19 is pro-obesogenic and enhances insulin resistance. Therefore, neutralisation of ADAM19 may be a potential therapeutic approach to treat obesity and T2D.

## 1. Introduction

Obesity is one of the most prevalent metabolic diseases in the Western world and correlates with glucose intolerance, insulin resistance, dyslipidemia, and cardiovascular disease. These metabolic parameters may ultimately culminate in pancreatic beta cell failure and type 2 diabetes (T2D) [1]. As the financial and social burden of obesity escalates, it is crucial to develop new human therapeutics to alleviate the adverse consequences of the metabolic syndrome.

There has been growing interest in the role of metalloproteinases or A Disintegrin and Metalloproteinase (ADAM) proteins, in metabolic disorders. ADAMs are proteolytic enzymes which regulate cell phenotype through affecting cell adhesion, migration, proteolysis, and signalling [2]. Our group was the first to identify that ADAM28 is elevated in humans with the metabolic syndrome and is a novel sheddase of human tumour necrosis factor-*α* (TNF-*α*) [3]. Relevantly, TNF-*α* is a major proinflammatory cytokine implicated in the metabolic syndrome [4, 5] and it is well established that TNF-*α* induces insulin resistance [6, 7]. Similarly, the activity of ADAM17, also known as TNF-*α* converting enzyme (TACE), correlates with insulin resistance [8, 9] and administration of TACE inhibitors to humans in clinical trial settings has proven to effectively decrease inflammatory mediators [10]. Interestingly, our group and others have shown that TNF-*α* is also a substrate for ADAM19 [11–13] and therefore it is possible that ADAM19 may have an underlying role in the pathogenesis of obesity and T2D.

ADAM19, also known as meltrin *β*, was identified and characterised by our team [14, 15] and others [16]. ADAM19 contains several complex domains, including a pro-domain, metalloproteinase domain, disintegrin domain, cysteine-rich domain, epidermal growth factor-like domain, transmembrane domain, and cytoplasmic tail domain [15]. Of most relevance, the metalloproteinase domain of ADAM19 is known to be involved in extracellular matrix breakdown and reconstruction [14]. One of the most important functions carried out by the metalloproteinase domain of ADAM19 is the catalytically mediated ectodomain shedding of substrates [14] such as TNF-*α*. ADAM19 is expressed in numerous metabolically relevant cell types and tissues, including peripheral blood mononuclear cells, liver, and skeletal muscle [14]. Furthermore, ADAM19 has been linked to numerous diseases [15] and serves important biological functions in embryogenesis [17], cardiovascular system development [18], and skeletal muscle adaptation [19]. Our study is the first to examine the role of ADAM19 in obesity, insulin resistance, and T2D.

In this study, we hypothesised that ADAM19 is associated with the metabolic syndrome in humans and mice. The outcomes of this study provide novel insights into the ADAM19-mediated pathogenesis of obesity, insulin resistance, and T2D.

## 2. Materials and Methods

### 2.1. Human Microarray for the Identification of Metalloproteinases Involved in Obesity and T2D

RNA samples (from peripheral blood mononuclear cells) were obtained from the San Antonio Family Heart Study (SAFHS) (*n* = 1240), a study of risk factors for cardiovascular disease in Mexican Americans living in and around San Antonio, Texas [20]. The SAFHS is a large family-based genetic epidemiological study including 1431 individuals from 42 extended families at baseline. Individuals from large randomly selected, multigenerational pedigrees were sampled independent of their phenotype or the presence or absence of disease. All participants in the SAFHS provided informed consent. The study and all protocols were approved by the Institutional Review Board at the University of Texas Health Science Centre at San Antonio (San Antonio, TX). Gene expression profiles were generated using microarrays, and data processing steps and quality control are as described previously [20].

### 2.2. Animals

In our first mouse cohort, eight-week-old male specific pathogen-free C57BL6/J mice were obtained from the Animal Resources Centre (ARC, Perth). Mice were administered a normal chow (14.3 MJ/kg, 76% of kJ from carbohydrate, 5% from fat, 19% from protein; Specialty Feeds, Glen Forrest, WA, Australia) or high fat diet, HFD (19 MJ/kg, 35% of kJ from carbohydrate, 42% from fat, 23% from protein; Specialty Feeds, Glen Forrest, WA, Australia) for 12 weeks. Body weights were recorded on a weekly basis. At the end of the experiment, mice were sacrificed and the liver and gonadal white adipose tissue was collected for paraffin embedding for immunohistochemistry and snap frozen in liquid nitrogen for mRNA studies.

In our second cohort of mice, eight-week-old male specific pathogen-free C57BL6/J mice were obtained from the Animal Resources Centre (ARC, Perth). Mice were placed on different diet/antibody treatment regimes: (1) standard chow (*n* = 9); (2) high fat diet, administered rabbit pre-immune IgG (*n* = 3); (3) high fat diet, administered rabbit ADAM19 neutralising antibody (*n* = 3). Neutralising antibodies were administered at week 10 of the dietary regiment as ADAM19 protein is increased at this time-point in tissues such as liver. Mice received 100 *μ*g of antibody every 2 days via tail vein for the final two weeks of feeding. Insulin tolerance tests (ITT; 0.5 U/kg) and glucose tolerance tests (GTT; 1 g/kg) were performed at the end of week 12. Body weight measurements were determined each week. Mice were sacrificed at the end of the experiment and their blood was collected for serum and livers were collected for paraffin embedding.

### 2.3. ADAM19 Neutralising Antibody

The rabbit ADAM19 neutralising antibody [pAb361] used for our experiments is well characterised by our team [21, 22].

### 2.4. ADAM19 Immunohistochemistry on Murine Liver

Liver tissue of mice fed with standard chow or HFD was fixed in 4% paraformaldehyde and paraffin embedded. Standard immunohistochemistry procedures were conducted, including antigen retrieval and incubations with primary antibody (rabbit anti-hADAM19 disintegrin domain IgG (pAb362)) at a 1 : 200 dilution [21, 23]. Sections were visualised with diaminobenzidine (DAB; DAKO).

### 2.5. *Adam19* and* Tnf-α* mRNA Expression Studies in Livers and/or Gonadal White Adipose Tissue of Mice Fed a HFD

RNA from livers of mice fed a HFD was extracted using Trizol reagent (Invitrogen) and cDNA synthesis was performed using the High Capacity RNA-to-cDNA kit (Applied Biosystems). Real-time PCR to determine the mRNA abundance of mouse* Tnf-α* and* Hprt* (house-keeper gene) was performed using a Rotor-gene real-time PCR machine (Qiagen) using predeveloped TaqMan probe (FAM labelled) and primer sets for mouse* Tnf-α* (Mm00443260_g1), mouse* Adam19* (Mm00477337_m1), and mouse* Hprt* (Mm01545399_m1) (Applied Biosystems). Quantitation was conducted as previously described [24].

### 2.6. TNF-*α* ELISA on Murine Liver Protein

Liver tissue was homogenised in cytosolic extraction buffer containing phosphatase and protease inhibitors. Protein levels were determined using a Bradford protein assay and TNF-*α* was measured in lysates using a mouse TNF-*α* ELISA (http://www.elisakit.com/).

### 2.7. Statistics

Associations between ADAM19 lymphocyte-derived gene expression and clinical parameters in the Mexican American pedigrees were determined using a linear mixed model allowing for residual non-independence amongst family members as a function of their genetic relationships. A likelihood ratio statistic was utilised to test whether ADAM19 expression significantly predicted a relevant standard clinical parameter (fasting insulin, BMI, HDL-C, relative fat mass, HOMA-IR index of insulin resistance, TNF-*α*, triglycerides, C-reactive protein, and IL-1*β*). Other primary covariates were simultaneously controlled for including sex and age. All analyses were performed using the statistical package, SOLAR.

All in vivo results are expressed as the mean + and/or − standard error of the mean (SEM). Data were analysed for differences by Student's *t*-test for unpaired samples where appropriate. Data was considered to be statistically significant when *p* ≤ 0.05.

## 3. Results

### 3.1. ADAM19 Expression in Human Blood Mononuclear Cells Is a Novel Marker of the Metabolic Syndrome

Here we use a large human cohort, the San Antonio Family Heart Study (SAFHS), which is well characterised for T2D and cardiovascular disease, that has also been extensively genetically analysed and carries full microarray-based genome wide gene expression profiles on 1,240 individuals. This dataset was filtered to identify novel metalloproteinases involved in the development of obesity and type 2 diabetes. We have found that high-level expression of ADAM19 in blood mononuclear cells from the SAFHS cohort correlated strongly with parameters of the metabolic syndrome and in particular BMI, relative fat, index of insulin resistance (HOMA-IR), and triglycerides (Table 1). In addition, ADAM19 expression in the SAFHS cohort also strongly correlated with circulating C-reactive protein, IL-1*β*, and TNF-*α* levels (Table 1). The latter is relevant as TNF-*α* is a substrate for ADAM19. These highly significant clinically relevant observations suggest a role for ADAM19 in the regulation of human metabolism.

### 3.2. ADAM19 and Its Substrate TNF-*α* Are Elevated in Liver Biopsies from Our HFD-Fed Mice

**Figure 1:**
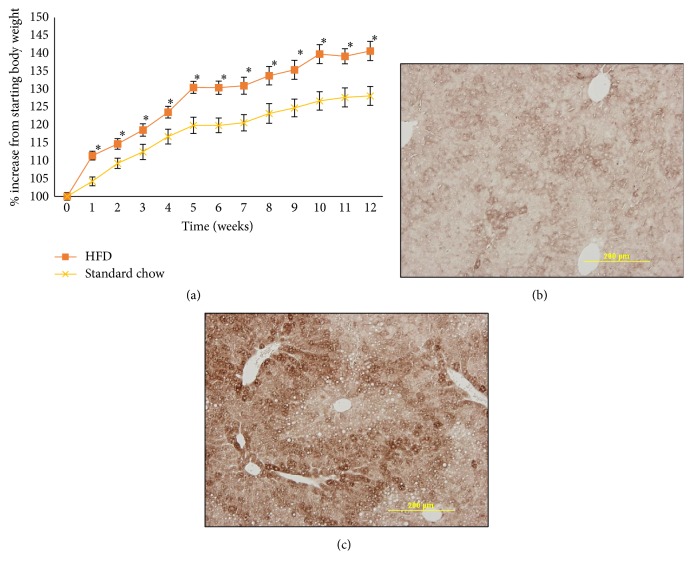
High fat diet induces obesity and elevates ADAM19 expression in mice. Mice fed a high fat diet gained weight (a); *n* = 14–18/group. Representative photomicrographs of ADAM19 protein expression in livers from mice fed either a normal chow (b) or high fat diet (c). ADAM19 staining is cytoplasmic and brown in colour. 200x magnification. ^*∗*^*p* < 0.05.

To complement our clinical observations, we explored ADAM19 expression and activity in animal models. Mice were weighed weekly to confirm obesity (Figure 1(a)). T2D was verified by insulin tolerance tests and glucose tolerance tests at the end of the experiment (Supplementary Figure 3 in Supplementary Material available online at https://doi.org/10.1155/2017/7281986) as previously conducted by our group [25]. We next investigated ADAM19 protein expression in liver, as it is an organ that is involved in lipid and glucose metabolism [26]. It has been previously reported that there is low expression of ADAM19 in murine liver [16]. We found that ADAM19 protein levels are increased in the highly steatotic livers of mice that have HFD-induced obesity and T2D compared to mice fed a normal chow (Figures 1(b) and 1(c)).

**Figure 2:**
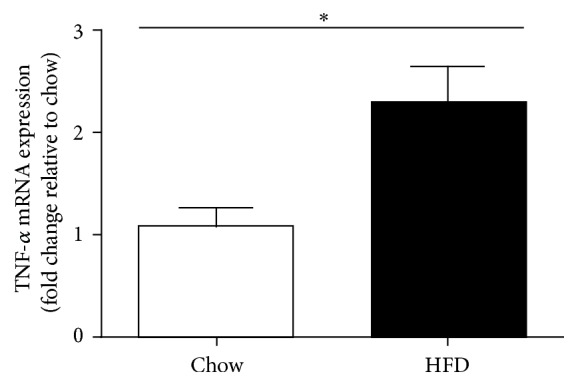
Elevated TNF-*α* mRNA levels in the livers of mice on a high fat diet for 12 weeks. ^*∗*^*p* < 0.05; *n* = 14–17 mice/group.

**Figure 3:**
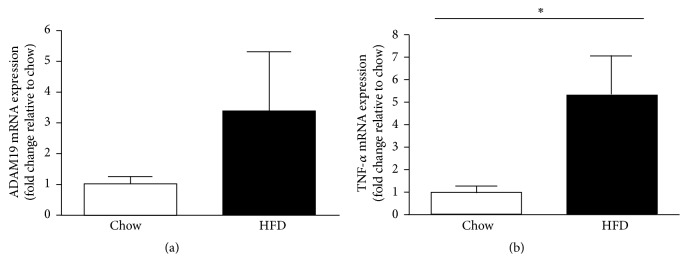
Elevated* Adam19* (a) and* Tnf-α* (b) mRNA levels in gonadal white adipose tissue of mice on a high fat diet for 16 weeks. ^*∗*^*p* < 0.05; *n* = 7-8 mice/group.

As previously mentioned, TNF-*α* is a proinflammatory cytokine involved in the metabolic syndrome and is a known substrate for ADAM19. Given that ADAM19 gene expression in human lymphocytes correlates with TNF-*α* protein level in the blood stream (Table 1) and ADAM19 protein is elevated in the liver of mice fed a HFD, we also wanted to determine whether* Tnf-α* gene expression is increased in the livers of our cohort of mice fed a HFD. We found that mRNA levels of* Tnf-α* are elevated in liver (Figure 2) biopsies from our mice that have HFD-induced obesity and T2D compared to chow fed mice. In addition, we also highlighted that both* Adam19* (Figure 3(a)) and* Tnf-α* (Figure 3(b)) gene expression were increased in gonadal white adipose tissue from HFD mice compared to chow fed mice.

### 3.3. Neutralising ADAM19 Causes Weight Loss in HFD-Fed Mice

**Figure 4:**
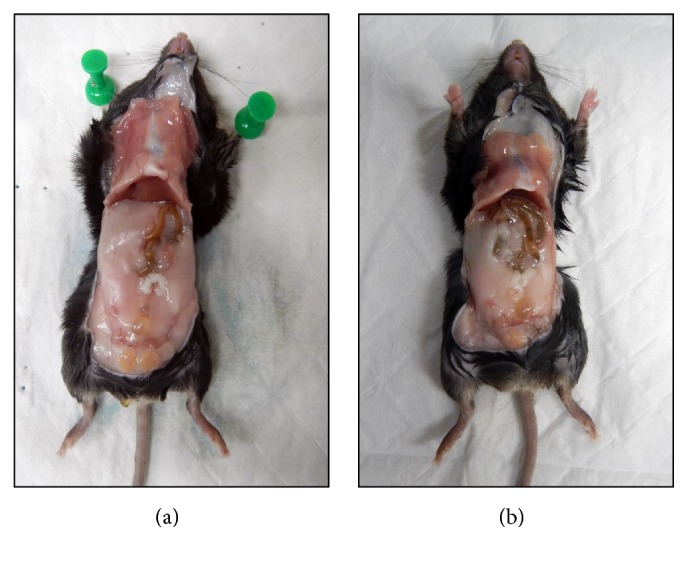
Neutralisation of ADAM19 reduces high fat diet induced obesity. Representative photographs of HFD-fed mice administered with either rabbit IgG (a) or ADAM19 neutralising antibody (b). Mice administered rabbit IgG possessed increased gonadal adipose tissue compared to mice administered ADAM19 neutralising antibody.

We are the first group to assess the effect of neutralising ADAM19 activity in vivo on the parameters of the metabolic syndrome. Through our ADAM19 neutralisation experiment, we found in our obese and T2D mice that neutralising ADAM19 activity minimises the accumulation of gonadal adipose tissue compared with mice administered the IgG control antibody (Figure 4).

**Figure 5:**
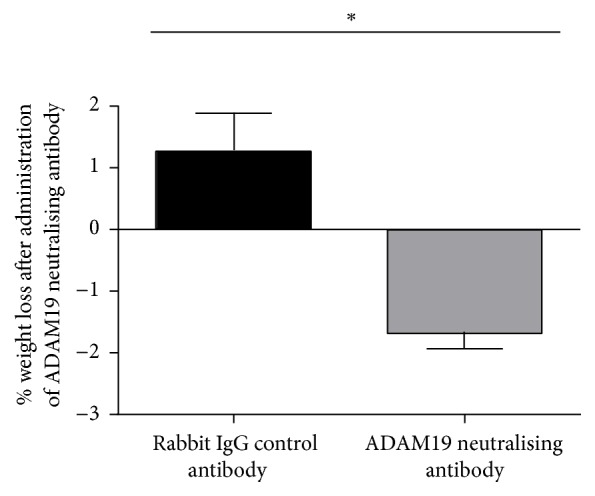
Elevated weight loss after administration of ADAM19 neutralising antibody. Mice which were administered ADAM19 neutralising antibody lost a significant amount of weight when compared with mice administered the rabbit IgG control antibody. Mean + SEM; ^*∗*^*p* = 0.0126; *n* = 3 mice/group.

Secondly, we showed for the first time that HFD-fed mice administered ADAM19 neutralising antibody lost a significant amount of weight when compared with mice that were administered rabbit IgG control antibody injections (*p* < 0.02) (Figure 5). Mice were monitored daily during antibody administrations and both groups displayed normal behaviour, posture, response to touch, and healthy coats. This novel finding provides evidence that neutralising antibody directed against the metalloproteinase domain of ADAM19 could be a potential therapeutic to alleviate symptoms of the metabolic syndrome.

### 3.4. Neutralising ADAM19 Improves Insulin Sensitivity in HFD-Fed Mice

**Figure 6:**
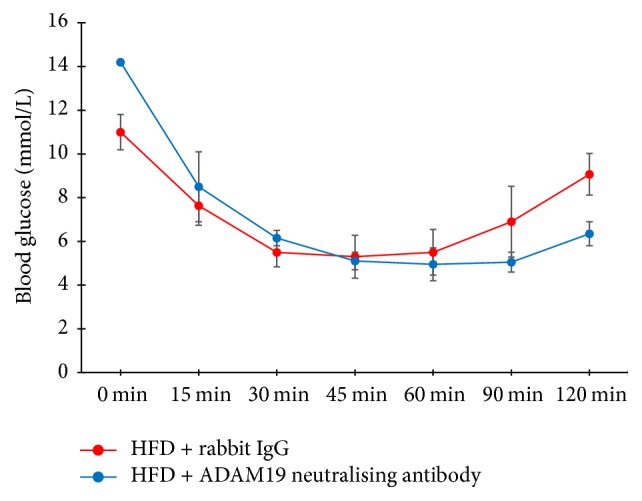
Neutralisation of ADAM19 improves insulin sensitivity in HFD-fed mice. Mean ± SEM; *n* = 3 mice/group.

Given that obese and T2D mice administered ADAM19 neutralising antibody lost weight, we were curious to explore if these mice were also more insulin sensitive. We performed an insulin tolerance test (ITT) at the end of the experiment. Despite the ADAM19 neutralising antibody group commencing the experiment with higher fasting glucose compared to the rabbit IgG group, the group administered with ADAM19 neutralising antibody had lower blood glucose levels by the conclusion of the experiment (Figure 6). This finding indicated that neutralising ADAM19 activity improved insulin sensitivity in HFD-fed mice, which correlated with weight loss. This novel data suggests that the ADAM19 metalloproteinase domain may play a crucial role in obesity, insulin resistance, and T2D.

### 3.5. Neutralising ADAM19 Reduces TNF-*α* Protein Levels but Does Not Effect ADAM19 Protein Expression in Livers from HFD-Fed Mice

**Figure 7:**
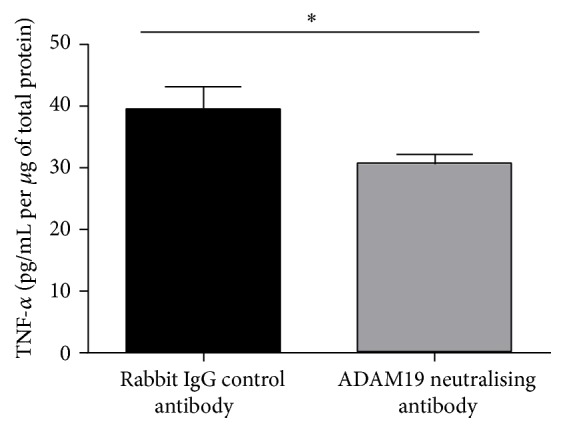
Neutralisation of ADAM19 reduces liver TNF-*α* levels in HFD-fed mice. Mean + SEM; ^*∗*^*p* = 0.05; *n* = 3 mice/group.

As ADAM19 is a sheddase of TNF-*α* protein, we measured the TNF-*α* protein levels by ELISA in the livers of mice treated with either rabbit IgG control antibody or ADAM19 neutralising antibody. We found that neutralising ADAM19 activity resulted in reduced TNF-*α* protein levels in the liver (Figure 7). The TNF-*α* protein may be cleaved or cytoplasmic derived TNF-*α*.

**Figure 8:**
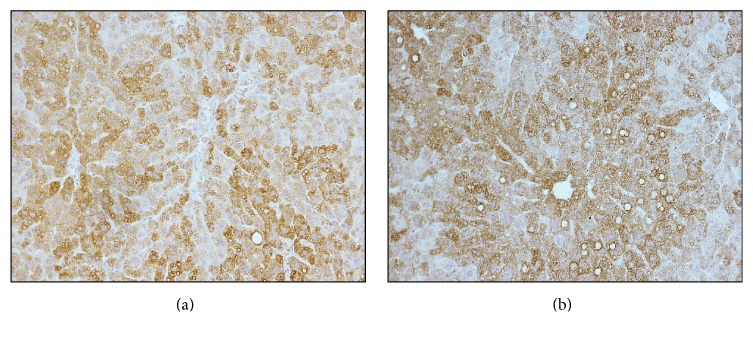
Neutralising ADAM19 does not affect the expression of ADAM19 in the liver. Representative photomicrographs showing ADAM19 immunohistochemistry performed on livers from HFD-fed mice administered either rabbit IgG control (a) or ADAM19 neutralising antibody (b). ADAM19 staining is cytoplasmic and brown in colour. 200x objective.

We also examined whether neutralising ADAM19 activity promoted ADAM19 expression to be elevated in a compensatory manner in livers from obese and T2D mice. We performed ADAM19 immunohistochemistry on livers of HFD-fed mice administered either rabbit IgG control antibody or ADAM19 neutralising antibody. We found that the level of expression did not differ between mice administered rabbit IgG control or ADAM19 neutralising antibody (Figure 8). Therefore, neutralising ADAM19 activity does not result in a compensatory upregulation of ADAM19 protein expression in the livers from obese and T2D mice.

### 3.6. In Vivo siRNA Mediated Knockdown of ADAM19

To support our insulin sensitivity findings from the ADAM19 neutralisation experiments, we then conducted experiments using siSTABLE siRNA targeting mouse ADAM19. We demonstrated that we could effectively knock down ADAM19 in vitro (Supplementary Figure 1) and in vivo (Supplementary Figure 2(A)). We highlighted that HFD-fed mice administered siSTABLE siRNA targeting ADAM19 possessed increased insulin sensitivity compared to HFD-fed mice administered non-targeting siSTABLE siRNA (Supplementary Figure 2(B)).

## 4. Discussion

Our group is the first to examine the role of ADAM19 in obesity and T2D. We found that high-level expression of ADAM19 in lymphocytes from the SAFHS cohort strongly correlated with the parameters of the metabolic syndrome. In particular, ADAM19 expression was significantly correlated with BMI, relative fat, HOMA-IR, and TNF-*α* levels. These significant and novel clinical observations provided strong support for a role of ADAM19 in the regulation of human obesity, insulin resistance, and T2D. This data prompted us to further evaluate the role of ADAM19 using our in vivo obese and T2D mouse model. We found that ADAM19 protein expression is elevated in highly steatotic liver and gonadal white adipose tissue of obese and T2D mice. This is extremely interesting as it has been previously reported that murine liver has low ADAM19 expression [16]. In our in vivo study, mice fed standard chow also had low ADAM19 expression.

We showed that TNF-*α* mRNA levels were significantly increased in liver and gonadal white adipose tissue of obese and T2D mice. TNF-*α* is a major proinflammatory cytokine implicated in the metabolic syndrome [4, 5], particularly insulin resistance [6, 7]. As TNF-*α* is a substrate for ADAM19 [11–13], we hypothesise that the shedding of TNF-*α* by the ADAM19 metalloproteinase domain potentially exacerbates the pathogenesis of the metabolic syndrome. In support of this hypothesis, we were able to demonstrate in our in vivo studies in HFD-fed mice that neutralisation of ADAM19 resulted in a marked reduction in TNF-*α* protein in the liver. This TNF-*α* protein may be cleaved or cytoplasmic derived.

We examined whether neutralising the metalloproteinase domain of ADAM19 would reduce the parameters of the metabolic syndrome in vivo. We are the first to show that neutralising ADAM19 activity reduces high fat diet induced obesity. In addition, neutralisation of ADAM19 promotes insulin sensitivity in obese and T2D mice. To support this finding, we demonstrated that HFD-fed mice administered siSTABLE siRNA targeting ADAM19 possessed increased insulin sensitivity compared to HFD-fed mice administered nontargeting siSTABLE siRNA. Combined, this novel data provides evidence that neutralising antibody directed against the metalloproteinase domain of ADAM19 could be a potential therapeutic to alleviate symptoms of the metabolic syndrome. Currently, there are no specific ADAM19 pharmacological inhibitors available and hence ADAM19 neutralising antibodies appear to be the most realistic available therapy to inhibit the function of ADAM19.

Targeting ADAMs with pharmacological inhibitors is being investigated as a potential therapeutic strategy for inflammatory diseases. The highly selective and orally active TACE (ADAM17) inhibitor, Ro 32-7315, has highlighted the reality that metalloproteinase inhibitors may inhibit inflammation induced TNF-*α* shedding and can be used safely in humans [27]. Of utmost importance is the fact that administration of TACE inhibitors to humans in clinical trial settings has proven to effectively decrease inflammatory mediators [10]. We used ADAM19 neutralising antibody in our current study which is a clinically relevant therapy. Neutralising antibody that targets TNF-*α* is currently used for rheumatoid arthritis in animal models and humans [28, 29]. Currently, there are a limited number of studies which utilise neutralising antibodies that target metalloproteinases in human disease [30–32]. Therefore, studies like ours are very important and may ignite further interest in the area.

Future studies should aim to examine potential upstream regulators of the increase of ADAM19 protein expression in relation to the metabolic syndrome in humans and mice. A study by Keating et al. (2006) identified that ADAM19 is induced by transforming growth factor beta 1 (TGF-*β*1) [33]. Human studies have shown that TGF-*β*1 is positively correlated with BMI [34] and increased plasma levels of TGF-*β*1 were reported in T2D [35]. Yadav et al. (2011) observed a significant correlation between TGF-*β*1 levels and adiposity in humans and rodents, and inhibiting TGF-*β*1 signalling protected mice from obesity, hepatic steatosis, and diabetes [36]. It would be intriguing to investigate the effect of TGF-*β*1 on ADAM19 expression and activation in our mouse model of obesity and T2D. Another interesting potential regulator of ADAM19 is furin. Our team previously reported that the protein furin activates ADAM19 by binding to and cleaving the prodomain of ADAM19 [37]. Relevantly, a polymorphism in the furin gene (rs17514846) has been shown to be significantly associated with hypertriglyceridemia and the metabolic syndrome [38, 39]. It would be insightful to ascertain whether furin protein levels are elevated in human patients with the metabolic syndrome and whether the binding of furin to ADAM19 pathogenically activates ADAM19 in vivo.

Whilst discussing potential regulators of ADAM19, it is relevant to also consider what transcriptionally controls ADAM19 expression in obesity and T2D. Putative binding sites for the transcription factors Sp1, Sp3, NF-kappaB, and VDR have already been identified in the ADAM19 gene [40]. If Sp1, Sp3, NF-kappaB, and VDR are increased in obese and T2D subjects, then this could be a mechanism underlying the elevated expression of ADAM19 in the metabolic syndrome.

## 5. Conclusion

Our exciting findings highlight that elevated ADAM19 protein expression is associated with the parameters of the metabolic syndrome in humans and mice. We identified for the first time that neutralising the ADAM19 metalloproteinase domain reduced high fat diet induced obesity and improved insulin sensitivity in obese and T2D mice. These novel results provide evidence that neutralising antibody directed against the metalloproteinase domain of ADAM19 could be a potential therapy for anti-obesity agents.

## Supplementary Material

Supplementary data includes methods and results pertaining to the mouse ADAM19 siRNA in vitro and in vivo studies as well as glucose tolerance testing data.

## Figures and Tables

**Table 1:** Parameters of the metabolic syndrome are associated with high ADAM19 expression in human peripheral blood mononuclear cells (*n* = 1240).

Parameter	*p* value	Direction of correlation
Fasting insulin	0.003	Positive
BMI	0.000046	Positive
HDL cholesterol	0.000065	Negative
Relative fat	0.000017	Positive
HOMA-IR	0.00014	Positive
TNF-*α*	0.018	Positive
Triglycerides	0.05	Positive
C-reactive protein	0.05	Positive
IL-1*β*	0.05	Positive

BMI: body mass index; HDL: high-density lipoprotein; TNF: tumor necrosis factor; IL: interleukin.
