# Supplementary material for: ADAM19: A Novel Target for Metabolic Syndrome in Humans and Mice
Source: Mediators Inflamm. 2017 Feb 7;2017:7281986. doi: 10.1155/2017/7281986 (PMC5318628; doi:10.1155/2017/7281986)
Supplement: Supplementary file 1 — Supplementary data includes methods and results pertaining to the mouse ADAM19 siRNA in vitro and in vivo studies as well as glucose tolerance testing data. [file 7281986.f1.pdf]

Supplementary Methods

In vitro knock-down of ADAM19

HEK293 cells were purchased from the American Type Culture Collection (Manassas, VA). HEK293 cells were cultured in DMEM (low glucose; Gibco) + 10% FCS and 1% penicillin/streptomycin at 37°C, 5% CO_2 in a humidified chamber. HEK293 cells were seeded into 6 well cell culture dishes and transiently transfected for 48hrs with a vector containing the cDNA for mouse ADAM19 (a kind gift from Professor Carl Blobel) with either 10nM of non-targeting siSTABLE siRNA (Dharmacon) or siSTABLE siRNA targeting mouse ADAM19 (Dharmacon) using Lipofectamine reagent (Life Technologies). ADAM19 expression in cells was determined by immunocytochemistry using rabbit anti-mouse ADAM19 polyclonal antibody (181-1; a kind gift from Professor Carl Blobel). Mouse ADAM19 mRNA levels were also determined using methods described in our manuscript.

ADAM19 Western blotting

Liver from mice was homogenised using cytosolic extraction buffer (10 mM hydroxyethyl piperazineethanesulfonic acid; 3 mM MgCl2; 14 mM KCl; 5% glycerol; 0.2% IGEPAL) containing phosphatase and protease inhibitors (Roche). Lysates were centrifuged at 13 000 rpm at 4°C for 10 minutes. Bradford assay (Bio-Rad, Hercules, CA, USA) was used to determine protein concentrations. Protein lysates (40 μg) were solubilized in Laemmeli sample buffer and boiled for 10 minutes, resolved by sodium dodecyl sulfate (SDS)–polyacrylamide gel electrophoresis on 10% polyacrylamide gels, transferred by semi-dry transfer to polyvinylidene difluoride membrane and blocked with 5% milk powder. Membranes were then incubated overnight at 4°C in primary antibodies [rabbit anti-hADAM19 metalloproteinase domain IgG (pAb361) or mouse anti β-actin (Abcam, Cambridge, UK; ab6276)] using recommended dilutions. Membranes were washed three times in washing buffer and incubated for 60 min at room temperature with either anti-rabbit or anti-mouse horse-radish peroxidase (HRP; Sigma, USA) respectively. Membranes were then washed and briefly incubated in Amersham ECL Prime Western Blotting Detection Reagent (GE). The protein bands were detected using the Alpha Innotech MultiImage II Fluor Chem FC2.

In vivo siRNA mediated knock-down of ADAM19

Eight week old male specific pathogen free C57BL6/J mice were fed a high fat diet for 10 weeks. At the end of week 10, mice were administered either non-targeted siSTABLE siRNA (n=3) or siSTABLE

mouse ADAM19 siRNA (n=3) for the final two weeks of feeding. We have chosen to administer siRNA at the end of week 10 of the dietary regiment as ADAM19 is increased at this time-point in tissues such as the liver. Mice received siRNA injections every 5 days via the tail vein. The siSTABLE siRNA (Dharmacon) have proven to be stable for greater than 5 days. For each injection, 20µg of siSTABLE siRNA was mixed with 200µl of DOTAP liposomal transfection reagent (Roche) and administered via the tail vein. Insulin tolerance tests were performed at the end of week 12.

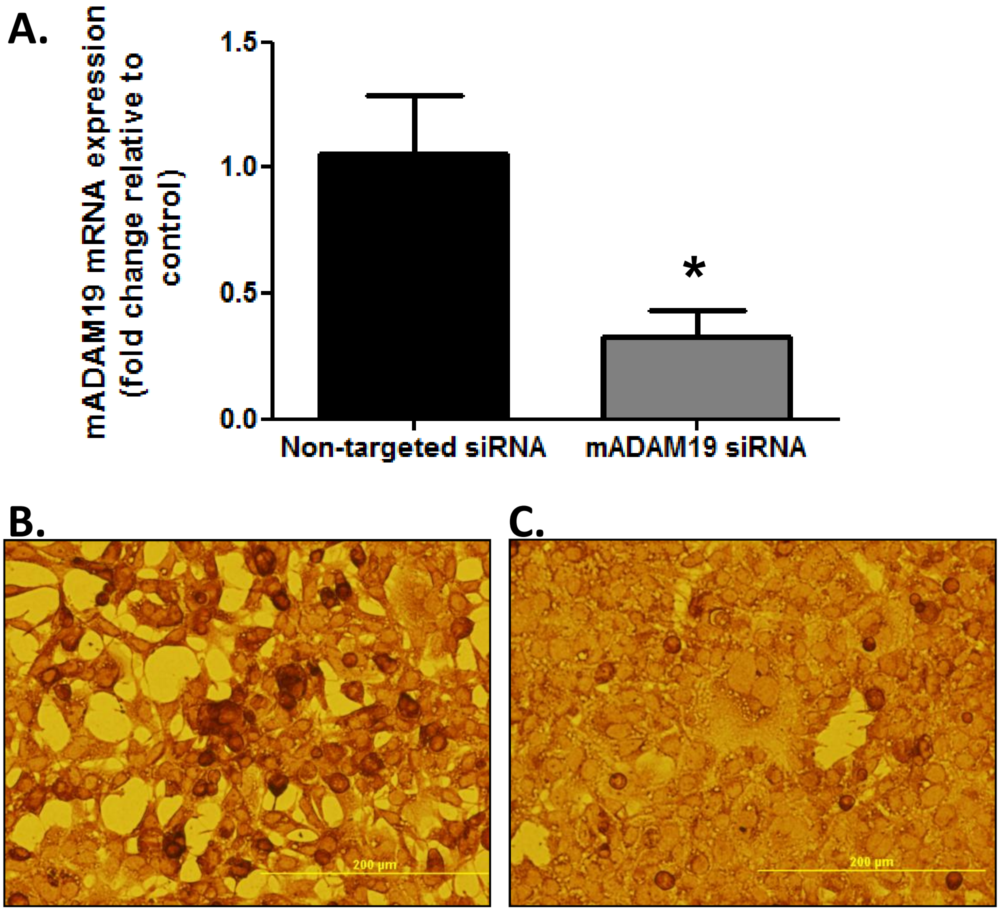

Supplementary Figure 1. Successful ADAM19 knock-down *in vitro* using siSTABLE siRNA. Mouse ADAM19 protein was overexpressed in HEK293 cells in conjunction with non-targeted siRNA or mADAM19 siRNA treatment. ADAM19 mRNA expression was knocked-down by siSTABLE siRNA in HEK293 cells (A). ADAM19 protein expression in HEK293 cells as determined by immunocytochemistry following (B) Non-targeted siRNA treatment and (C) mADAM19 siRNA treatment. 100x magnification; *p<0.05.

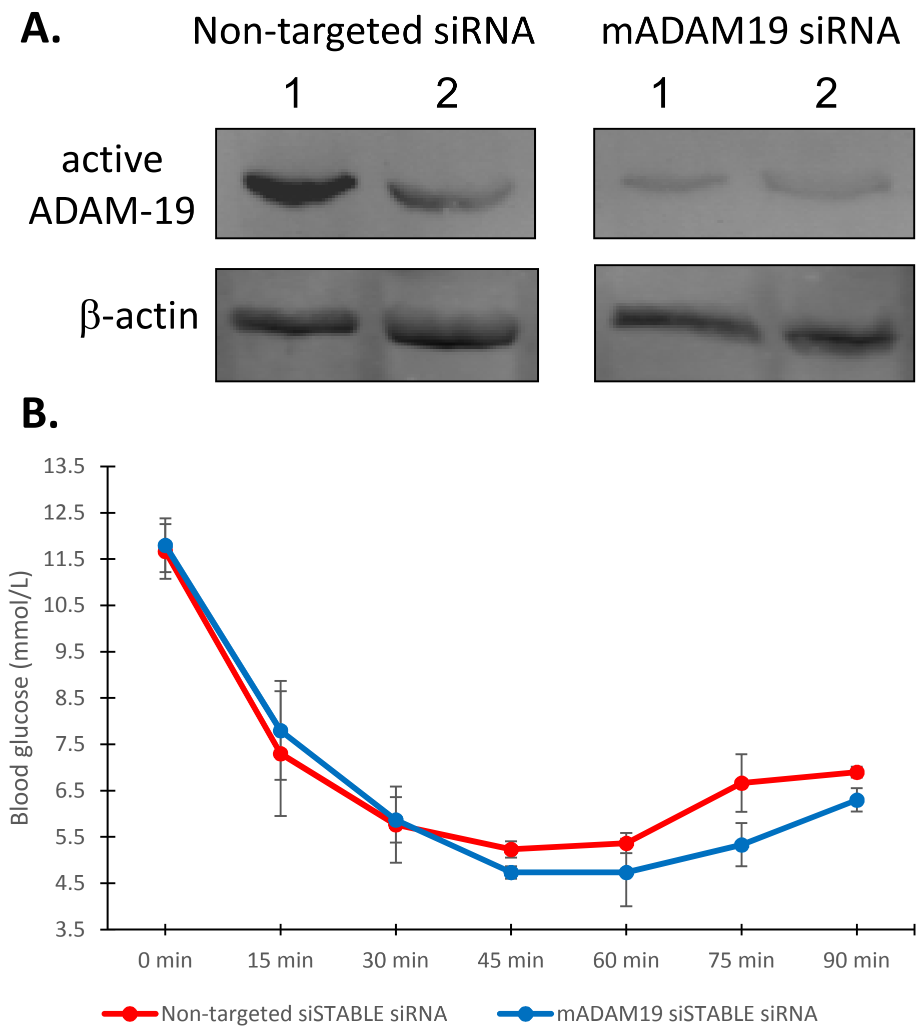

Supplementary Figure 2. Successful ADAM19 knock-down in liver of mice on a high fat diet using siSTABLE siRNA *in vivo*. (A) Western blotting for ADAM19 protein in liver of mice fed a HFD following non-targeted siRNA or mADAM19 siRNA treatments. β-actin was used as a control. (B) Knock-down of ADAM19 improves insulin sensitivity in HFD-fed mice. Mean ± SEM; n=3 mice/group.

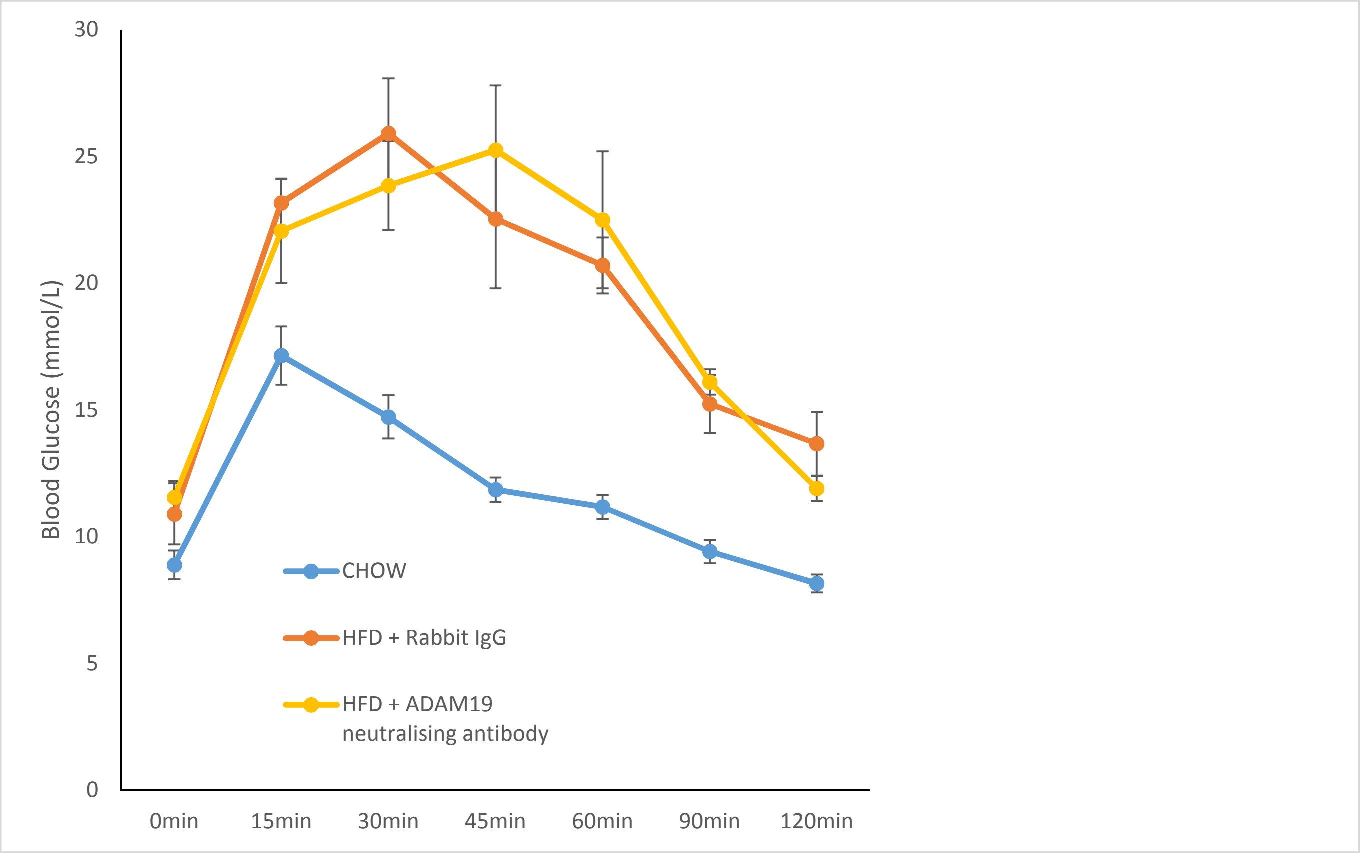

Supplementary Figure 3. Mice fed a HFD display glucose intolerance compared to mice fed normal chow.
